# An analysis of the potential for achieving the fourth millennium development goal in SSA with domestic resources

**DOI:** 10.1186/s12992-015-0092-1

**Published:** 2015-02-25

**Authors:** Bernadette O’Hare, Innocent Makuta

**Affiliations:** College of Medicine, University of Malawi and University of St Andrews, Scotland. 360, Chichiri, Blantyre 3 Malawi; Department of Economics, Chancellor College, University of Malawi, Zomba, Malawi; Department of Business Administration, Malawi Polytechnic, Blantyre, Malawi

**Keywords:** Illicit financial flows, Corruption, Debt, Under-five mortality, Sub Saharan Africa, Millennium development goals

## Abstract

**Background:**

The importance of good health is reflected in the fact that more than half of the eight Millennium Development Goals (MDGs) are aimed at improving health status. Goal 4 (MDG4) aims to reduce child mortality. The progress indicator for goal 4 is the under-five mortality rate (U5M), with a targeted reduction of two thirds by 2015 from 1990 levels. This paper seeks to compare the time (in years) Sub Saharan African (SSA) countries will take to reach their MDG4 target at the current rate of decline, and the time it could have taken to reach their target if domestic resources had not been lost through illicit financial flows, corruption and servicing of debt since 2000.

**Methods:**

We estimate the amount by which the Gross Domestic Product (GDP) per capita would increase (in percentage terms) if losses of resource through illicit financial flows, corruption and debt servicing, were reduced. Using the income elasticity of U5M, a metric which reports the percentage change in U5M for a one percent change in GDP per capita, we estimate the potential gains in the annual reduction of the under-five mortality if these resource losses were reduced.

**Results:**

At the current rate of reduction in U5M, nine countries out of this sample of 36 SSA countries (25%) will achieve their MDG4 target by 2015. In the absence of the leakages (IFF, corruption and debt service) 30 out of 36 (83%) would reach their MDG4 target by 2015 and all except one country, Zimbabwe would have achieved their MDG4 by 2017 (97%). In view of the uncertainty of the legitimacy of African debts we have also provided results where we excluded debt repayment from our analysis.

**Conclusions:**

Most countries would have met MDG4 target by curtailing these outflows. In order to release latent resources in SSA for development, action will be needed both by African countries and internationally. We consider that stemming these outflows, and thereby reducing the need for aid, can be achieved with a more transparent global financial system.

## Background

Improved health is not only a goal in its own right but also an ingredient for accelerated attainment of sustainable development [1]. The importance of good health is reflected in the fact that more than half of the eight Millennium Development Goals (MDGs), agreed upon by world leaders in 2000 at a United Nations summit, are aimed at improving health status of the poor. The MDGs are a time-bound set of eight goals aimed at improving the conditions of living for the world’s poorest by 2015. They include 18 targets and 48 progress indicators that, among other things, monitor both upstream root causes of poverty and poor health, and the downstream health outcomes such as child mortality.

Goal 4 (MDG4) aims to reduce child mortality. The progress indicator for MDG4 is the under-five mortality rate (U5M), with a targeted reduction of two thirds by 2015 from 1990 levels. Child mortality is an indicator not only of the state of children’s health but also of the efficacy of a government’s social policies [2]. Sub-Saharan Africa (SSA) suffers the highest U5M with mean child mortality rate at 98 per 1000 live births in 2012 [3]. SSA has more than doubled its average rate of reduction of U5M from 1.5% per year in the period 1990–2000 to about 4.0% per year for the period 2000–2012 [4]. However, this pace of progress is insufficient to achieve the MDGs by the target date of 2015. Indeed, using the annual rate of reduction for all the individual SSA countries between 2000 and 2011, it has been estimated that only six out of 34 SSA countries in this region will achieve their MDG4 target by 2015 [5].

A major obstacle to progress on meeting the MDG targets in SSA, including MDG4, has been the lack of financial resources. Notwithstanding this fact, the region has been a “net creditor” to the rest of the world as a result of illicit financial flows (IFF) [6]. It is against this background of slow progress on the MDGs, amid massive leakages of resources that this paper intends to examine effect of loss of financial resources from SSA on the progress on MDG4.

### Aims

This paper seeks to compare the time (in years) SSA countries will take to reach their MDG 4 target at the current rate of decline and the time it could have taken to reach their target if domestic resources had not been lost through illicit financial flows, corruption and servicing of debt since 2000.

### MDG Financing gaps and lost resources: what is known?

At the onset of the MDG period, a High-Level Panel on Financing for Development foresaw the shortfall in the capacity of developing countries to mobilise domestic resources required to meet the MDG targets and recommended that the cost of financing the MDGs be shared by rich and poor countries. A call was made by leaders of the UN Millennium project for a “big push” of increased and predictable financial support from high income countries, as poor countries could not reach these health and social goals because they were simply too poor [7]. Several estimates were made of the global annual financial gap, measuring the difference between the estimated total cost and the resources available from domestic sources. These estimates ranged widely depending on the methodology used and assumptions made, from around $50 billion to the highest estimate at $195 billion of overseas development aid required each year [8]. The overall cost of achieving MDG 4 and 5 (those related to child mortality and maternal health respectively) in SSA was estimated to be about $63.2 billion of which $27.5billion is an annual financial gap. Table 1 summarises some of these estimates.

**Table 1:** **Estimates of the cost of achieving the MDGs**

**Organisation providing the estimate**	**Annual financing gap** (**USD)**	**Scope**
High-level Panel on Development (UN, 2002)	50bn	All goals, all regions
World Bank (2002).	40bn – 60bn	All goals, all regions
United Nations (2005)	52bn - 73bn (in2006) and 110 - 135bn (in 2015)	All goals, all regions
Commission for Macroeconomics and Health	27bn – 38bn	Goals 4,5 and 6 Health sector only, all regions
Commission for Macroeconomics and Health	27.5bn (35.7bn can be mobilised domestically)	Goals 4,5 and 6 SSA only
UNDP (Building on Evidence: Corruption a Major Bottleneck to MDG 2012)	30bn	Eradicate hunger, all regions
	18bn	To provide improved water and sanitation, all regions

As pointed out earlier, a major obstacle to funding the MDG in SSA from domestic sources has been lack of financial resources, yet the region has been a “net creditor” to the rest of the world as a result of illicit financial flows (IFF) and debt service. Authoritative analyses have demonstrated that IFF have driven capital out of Africa far in excess of capital inflows of Foreign Direct Investment (FDI) and aid [6]. It has been estimated that SSA loses twice as much through IFF than it gains through official development assistance [9]. IFF are defined as funds that are illegally earned, transferred or utilised and include all unrecorded private financial outflows that drive the accumulation of foreign assets in contravention of applicable laws [10]. IFF occur through tax evasion, corruption and laundering the proceeds of criminal activities, the majority of which is a result of efforts to evade tax by multinational enterprises (MNE). It is estimated that 78 percent of all IFF from developing countries are attributable to trade mis-invoicing by MNE [11]. The African Development Bank estimated that Africa has lost $1.4 trillion from 1980 – 2009, and this outflow has been escalating since 2000 [6], increasing at a rate of 9 percent per year [11]. It is important to note that this estimate for IFF does not include resources lost due to corruption.

Corruption is defined as the abuse of public office for private gain [12] and remains a major challenge in Africa as it does in wealthy countries [13]. The available estimates of losses due to corruption in Africa vary, but the most widely quoted estimate is 25% of GDP per year (about $148 billion) by the African Union [14]. Corruption affects child health, and therefore child mortality, by two main channels; the impact on economic growth which will impact household income and the impact on the provision of public services. It has been shown that a unit increase in the corruption perception index leads to a loss of growth of per capita GDP of 0.59 percentage points [12]. The pathway by which corruption may affect the provision of public services results from the increase in price for these services which reduces the quantity and quality of services available. The effect on economic growth also reduces government revenue, which lowers the quality of public services which can be afforded. Poor quality services are a disincentive for people who can afford private services and reduces their willingness to pay tax, thus reducing the tax base [15].

Some loans to African countries have funded development but some have not. Ndikumana and Boyce have analysed the relationships between debts and capital flight and found that 50% of capital loaned to African countries left those countries the same year as capital flight. Debts which did not benefit the population, about which they had no knowledge and in situations where the creditors were aware of the circumstances, are defined as odious in international law [16]. Since the 1970s, African public debt has piled up, as foreign loans were diverted into private assets abroad. In 2010, African debt stood at $300 billion with an annual debt service of $22 billion [17]. What is unclear is the proportion of Africa’s debt that can be described as odious. In view of the controversy over the legitimacy of African debts we provided estimates which include the servicing of debt and estimates where debt service is not included.

### Lost Resources and Child Mortality: the Pathways

There are two primary channels through which lost resources will translate into slower progress in reducing child mortality: reduced income and lower public provision of services. In the reduced income channel, resource flight represents lost opportunities for investment and income-generation. A country therefore realises lower GDP per capita, which means that on average, households will command fewer resources. A negative income-child mortality association has been firmly established. The landmark paper ‘Wealthier is Healthier’ showed using instrumental variables that the relationship between income and health is not only associative but causative and suggested that increased income improves health by expenditure on goods which improve health or the socioeconomic determinants of health [18]. The importance of income has been confirmed by many others and summarised in a systematic review [19]. In the present case this lower income, due to resource outflows, translates to a slower reduction of child mortality.

In the public service provision channel, the lost resources represent lost government revenues. IFF reduces the tax revenue as they involve tax evasion. Debt service and some forms of corruption compete for the same resources as public provision of social services. Therefore, the available resource envelope at the disposal of a government is reduced and the proportion spent on providing social services is also reduced.

**Figure 1:**
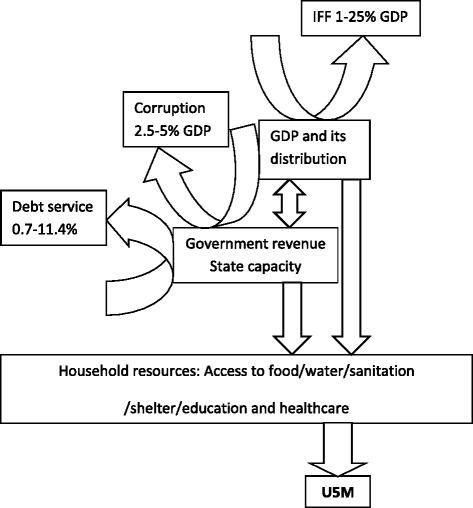
**Relationship between resources and U5M.** Adapted from [5] with permission.

The pathways through which GDP may affect child mortality (reduced household income and lower public provision of services), and thus time to reach the MDG4, is represented schematically in Figure 1.

The relative importance of these two channels has not been established and it is likely that the balance varies between countries and between different aspects of child welfare. For example, when looking at childhood deprivation in 68 low and middle income countries, GDP per capita was found to be more important than quality of governance for shelter, sanitation and education. However, quality of governance was more important than GDP per capita for provision of safe water, malnutrition prevention and healthcare [20]. There are also different aspects of governance: it has been asserted that “the rule of law influences child mortality more than the size of the government’s coffers” [21] and some governments have been considered to be better than others at converting resources into human development [22]. In the absence of state capacity to provide public goods such as water and sanitation, families have to provide these and will be in a better position to do so if their household income allows. It is therefore likely that in countries with low state capacity the direct impact on household income predominates over the impact of the state on service provision and that corruption through its impact on economic growth [12] has an impact on child mortality predominantly via household income rather than via the services provision pathway.

## Methods

We estimate the amount by which the current GDP per capita would increase (in percentage terms) if losses of resource through illicit financial flows, corruption and debt servicing, were reduced. To compute these gains in GDP per capita, we rely on previous work by Global Financial Integrity (GFI) [23] where IFF lost were expressed as a percentage of GDP. These IFF/GDP ratios can also be interpreted as per capita ratios. We take the approach that these IFF/GDP ratios figures represent the amount by which GDP per capita in the country of origin would have increased if the resources had not leaked out. However, it is probable that the GDP would have risen to a greater extent than this estimate given the investment-multiplier effects. Therefore, the estimates presented here are conservative.

Ugur and Dasgupta synthesised 115 studies on the effect of corruption on economic growth. In this meta-analysis, they found that a unit increase in the corruption perception index leads to a loss of growth of per capita GDP of 0.59 percentage points [12]. We use this estimate to calculate the potential growth of per capita GDP achievable in the absence of any perception of corruption using Transparency International’s index. The Transparency International Corruption Perception Index (CPI) ranges from 0 (the most corrupt) to 10 (the least corrupt) [24]. A majority of African countries have a CPI of less than four. We have estimated the potential economic growth that would have been attained if each of these countries had maintained a CPI of 10 since the year 2000.

We assume that the allocation of government resources does not change and that each sector including the health sector continues to receive the same proportion of funding with the increased availability of resources as corruption and IFF are reduced. This assumption is reasonable given the evidence that reduced corruption, will lead to economic growth [12] and improved governance results in increased spending on, and provision of, social services include health care services [15].

For figures on debt service, we use data from the World Bank’s World Development Indicators series. We then sum the losses of resource through IFF, corruption and debt service to get the overall annual loss of resources (as a percentage of GDP per capita).

Next, we estimate the potential gains in the annual reduction of the under-five mortality. In order to do this we multiply the percentage by which the GDP per capita would increase, by the income elasticity of U5M, a metric which reports the percentage change in U5M for a one percent change in GDP per capita. The pooled estimate for SSA, derived after systematic review and meta-analysis, is 0.38 [19].

To derive the number of years it would take to reach MDG 4 under two scenarios, with and without resource leakages, we use the following formula:

1 $$ F=P{\left(1-r\right)}^n $$

Where F is the future (or desired) under-five mortality rate, P is the actual (2000) under-five mortality rate, *r* is the rate of decline in under-five mortality rate and *n* is the number of years

Solving for n in (1), we have:

2 $$ n=\frac{ \ln \left(F/P\right)}{ \ln \left(1-r\right)} $$

We calculate actual n (in the presence of resource leakages) using the actual rates of reduction [4], as r. To compute n in the absence of resource leakages, we proceed as follows: losses to IFF and debt service are treated as realisable increases in GDP per capita if these losses did not occur. For corruption, we assume absence of corruption by taking a score of 10 on Transparency International’s index, i.e. it is perceived that there is no corruption. We then calculate the annual increase in per capita GDP by taking the difference between each country’s CPI score and 10 and then taking the multiplying this difference with 0.59 from the meta-analysis cited above. We then aggregate the potential increase in GDP per capita from curtailing these resource outflows. The potential increase in rate of reduction of U5M is calculated by taking the product of increase in GDP per capita and the income-child mortality elasticity of 0.38. The GDP per capita/under five-mortality elasticity is based on the increase in economic growth which comes when there is no perceived corruption and our model assumes that each sector including the health sector continues to receive the same proportion of funding with the increased availability of resources as corruption and IFF are reduced.

## Results

### Country level estimates for gains in U5M decline

We have provided estimates on 36 countries out of a total of 48 in the region because for some countries no estimate of their IFF was available. We have estimated the year when the MDG for each country will be reached at the current rate of decline using the most recent estimates (2000–2012) and we have estimated the year that countries will reach their MDG target if domestic resources had been fully mobilised or losses of resources through illicit financial flows, corruption and debt servicing, were curtailed.

As Table 2 shows, at the current rate of reduction in U5M, nine countries out of this sample of 36 SSA countries (25%) will achieve their MDG4 by 2015 (column 11). In the absence of the leakages (IFF, corruption and debt service) 30 out of 36 (83%) would reach their MDG by 2015 and all except one country (Zimbabwe) would have achieved their MDG by 2017 (column 13). In view of the uncertainty of the legitimacy of African debts we have excluded debt repayment from one of our analysis (column 12). However we note that this has little influence on our findings in terms of years to reach the MDG.

**Table 2:** **The impact of curtailing the leakage of resources on time to reach MDG4 in SSA**

**Country**	**2000 Actual mortality rates (2014 report)**	**MDG goals**	**Actual rates of reduction (%)**	**Non-normalised IFF (%of GDP)**	**Corruption perception index (Transparency International)**	**Percentage of GDP lost corruption**	**Potential rates of reduction (IFF and Corruption)**	**Debt service (% of GDP)**	**Potential rates of reduction (IFF, Corruption and debt service)**	**Number of years to reach MDG4 (at actual rates)**	**Number of years to reach MDG4 (potential rates-IFF and CORPN)**	**Number of years to reach MDG4 (potential rates- IFF,COPRN and debt)**
Angola	203	87	1.8	7	1.9	4.8	6.28	11.4	10.61	47	13	8
Benin	174	61	4.2	1	2.9	4.2	6.17	1.2	6.63	24	16	15
Botswana	85	17	3.9	10	5.7	2.5	8.66	0.7	8.93	40	18	17
Burkina Faso	186	67	5.0	3	3.4	3.9	7.62	1	8.00	20	13	12
Burundi	150	63	3.0	6	1.8	4.8	7.12	2.5	8.07	28	12	10
Cameroon	150	50	3.8	6	2.4	4.5	7.78	3	8.92	28	14	12
Central African Republic	164	59	2.0	5	2.0	4.7	5.69	1.5	6.26	51	17	16
Chad	189	67	1.9	20	1.6	5.0	11.38	1.7	12.03	54	9	8
Congo, DR	171	66	1.3	3	2.0	4.7	4.23	4.7	6.02	73	22	15
Congo, Rep	118	35	1.7	25	1.9	4.8	13.02	2.5	13.97	71	9	8
Cote d’Ivoire	145	51	2.5	6	2.1	4.7	6.55	4.4	8.22	41	15	12
Ethiopia	146	70	6.3	6	2.7	4.3	10.22	1	10.60	11	7	7
Gabon	86	31	2.7	11	2.9	4.2	8.47	7.2	11.21	37	12	9
Gambia	116	55	3.9	14	2.7	4.3	10.86	3.5	12.19	19	6	6
Ghana	103	39	3.0	2	4.0	3.5	5.11	3	6.25	32	19	15
Guinea	171	76	4.4	9	1.8	4.8	9.66	4.1	11.22	18	8	7
Guinea Bissau	174	80	2.5	7	2.0	4.7	6.95	1.7	7.60	31	11	10
Kenya	110	35	3.4	1	2.1	4.7	5.55	2.5	6.50	33	20	17
Lesotho	114	34	1.1	15	3.3	4.0	8.30	3.7	9.71	109	14	12
Madagascar	109	56	5.2	6	2.8	4.2	9.09	1.2	9.55	12	7	7
Malawi	174	75	7.5	10	3.2	4.0	12.82	1.7	13.47	11	6	6
Mali	220	83	4.5	3	2.9	4.2	7.23	1.8	7.92	21	13	12
Mauritania	111	43	2.3	12	2.5	4.4	8.54	3.5	9.87	41	11	9
Mozambique	166	83	5.1	5	2.6	4.4	8.66	1.3	9.15	13	8	7
Niger	227	102	5.8	3	2.8	4.2	8.55	1.3	9.05	13	9	8
Nigeria	188	77	3.5	12	2.5	4.4	9.74	2.8	10.81	25	9	8
Rwanda	182	58	10.0	5	3.4	3.9	13.38	0.9	13.72	11	8	8
Senegal	139	50	7.1	1	3.1	4.1	9.03	3	10.17	14	11	10
South Africa	74	19	4.2	4	4.7	3.1	6.91	2.4	7.82	32	19	17
Sudan	106	41	3.1	3	1.5	5.0	6.15	1.3	6.64	30	15	14
Swaziland	121	28	3.4	11	3.5	3.8	9.04	1.5	9.61	42	15	14
Tanzania	132	52	7.4	2	2.8	4.2	9.77	0.9	10.12	12	9	9
Togo	122	50	2.0	6	2.6	4.4	5.94	1.8	6.62	44	15	13
Uganda	147	62	6.3	3	2.5	4.4	9.12	1	9.50	13	9	9
Zambia	169	57	5.4	9	2.9	4.2	10.41	4.4	12.08	20	10	8
Zimbabwe	102	26	1.1		2.1	4.7	2.87	3.7	4.28	124	47	31
Total	146	56	4.0			5.9	6.19		6.19	24	15	15

## Discussion

### Principal findings

The leakage of resources out of SSA has greatly impeded progress in meeting MGD4. These findings are consistent with our previous projections, that only six countries out of 34 in SSA will achieve the MDG4 targets by 2015, against sixteen which would do so in the absence of IFF [5]. The estimate has increased from six out of 34 to nine countries out of 36 reaching their MDG4 by 2015 because we use more recent estimates which suggest a slightly faster current rate of decline. This paper adds to these previous findings by considering progress that would have been attainable in the absence of corruption and the burden of debt service, in addition to IFF. The results add further weight to the widely accepted argument that corruption and tax evasion have a negative impact on economic development because they divert public resources for private use and erode the government’s tax base, ultimately impeding efforts to reduce poverty [25]. The present findings provide evidence of the impact of these mechanisms using the specific example of child mortality. There is little precedent in the literature of the effects of IFF and corruption on health outcomes in terms of number of years to reach MDG4 targets. A limitation of this analysis is that we were able to include only 36 countries owing to data constraints. Further, perceptions of corruption are not entirely objective and therefore our results are not conclusive but nonetheless indicative of likely impacts.

#### IFF

Domestically mobilised resources including taxation are generally recognised as the most predictable source of finance for governments and since the financial crisis of 2008, this has become a priority for financing the MDGs [26]. Average tax revenues as a proportion of GDP are generally low in low-income countries; half of the countries in SSA collect less than 17% of their GDP in tax as compared to high income countries where the average is 35% [27]. SSA loses up to half of its potential corporate income tax revenue, which is a major component of total government revenue in low income countries, as a result of IFF [28]. It is therefore not surprising that previous research on IFF and child mortality have shown a negative association, a message similar to that presented in this paper. For example Christian Aid [29] estimated tax losses due IFF may be responsible for 5.6 million child deaths between 2000 and 2015. Similar results have been obtained by Save the Children [30] which estimates that if developing countries were to reduce IFF more child deaths could be avoided each year and all preventable child deaths could be avoided twenty years ahead of current predictions, which are presently at 2050. The OECD has called for policy coherence for development with respect to IFF and the role OECD countries play in facilitating these [31].

#### Corruption

In 2004 the World Bank estimated that countries which tackle corruption can increase their per capita incomes by 400% [32]. The allocation of government contracts through a corrupt system will result in poor quality infrastructure and services, including health and education [33], which will impact child welfare and therefore mortality. Kauffman and colleagues found a strong causal effect between improved governance and better developmental outcomes. They examined 300 measures of governance and found strong agreement across the many different indexes of the quality of governance. They found that a standard deviation increase in any of their six measures of governance was associated with a 2.5 fold increase in per capita GDP, a fourfold decrease in infant mortality and a 15-25% increase in literacy [34]. Gupta and colleague find that child mortality is one third higher in corrupt versus less corrupt countries, infant mortality and low birth weight is twice as high and school drop put is five times higher in corrupt countries. They find that the effect of social spending on health and education is largely insignificant except that public education spending may reduce the dropout rates in less corrupt countries. They find that income elasticity of child mortality in countries with low corruption is twice as high as countries with high corruption, which suggests that income is more effective at improving social indicators in countries with low corruption [15]. Recent work confirms that social spending has more impact in well governed countries [35]. In this work we used the per capita GDP channel to quantify the impact of corruption on child mortality and the present findings confirm and further quantitate the extent of negative health effects of corruption in terms of lost years to achieve MDG4. It is likely that the impact is via the household income, and therefore would not be influenced by decisions with regards to the allocation of resources; nonetheless our model assumes that each sector including the health sector continues to receive the same proportion of the increased funding available.

#### Debt

Discriminating between odious and legitimate debts requires auditing. African countries are likely to benefit from debt audits and repudiation of odious debts but leaders who have benefited personally from irresponsible borrowing are unlikely to initiate such audits. Given that half of African loans left in the form of capital flight it is likely that at a minimum 50% of African debt is odious but this requires auditing and repudiation at national level [16]

## Conclusions

Given the massive drainage of resources through IFF, the findings that SSA would have achieved more in reducing child mortality and most countries would have met MDG4 target by curtailing these outflows are unsurprising. In order to achieve such curtailment, action will be needed both by individual African countries and internationally mainly in OECD countries. We consider that IFF can be curtailed by poor and rich countries working together to create a more transparent global financial regime that limits financial secrecy and legislates for companies to report profits according to the country in which they were made.

Corruption is another important way in which resources are lost. Corruption stagnates economic growth [36] and will impact child survival via the effect on household income and on the efficacy of social spending. Measures that have been shown to reduce corruption should be taken, including decentralisation of budgets, community participation and transparency in contracts [37]. Western countries and multinational enterprises can facilitate this effort by publishing in an accessible format their overseas development aid, contracts agreed to and country by country reporting of profits. African countries should audit their debt to determine legitimacy; they could be greatly helped in this endeavour by their development partners who could take the initiative to audit their debts.

Regardless of how good plans for the post 2015 development agenda may be, SSA is likely to continue to lag behind and miss proposed targets if the plans do not incorporate serious mechanisms of curtailing IFF, corruption and servicing of illegitimate debt. It is imperative that domestic resource mobilisation be strengthened to improve the progress on reducing child mortality and the achievement of other development goals.

## Abbreviations

CPI: Corruption perception index
FDI: Foreign direct investment
GDP: Gross domestic product
IFF: Illicit financial flows
MDG: Millennium development goal
MDG4: The fourth millennium development goal
OECD: Organisation for economic cooperation and development
SSA: Sub Saharan Africa
U5M: Under-five mortality
